# Unlocking the Potential of Bioactive Compounds in Pancreatic Cancer Therapy: A Promising Frontier

**DOI:** 10.3390/biom15050725

**Published:** 2025-05-15

**Authors:** Silvia Brugiapaglia, Ferdinando Spagnolo, Claudia Curcio

**Affiliations:** 1 Department of Molecular Biotechnology and Health Sciences, Piazza Nizza 44b, 10126 Turin, Italy; silvia.brugiapaglia@unito.it; 2 School of Advanced Defence Studies, Defence Research & Analysis Institute, Piazza della Rovere 83, 00165 Rome, Italy; ferdinando.spagno57@edu.unito.it; 3 Defense Institute for Biomedical Sciences, Via Santo Stefano Rotondo 4, 00184 Rome, Italy

**Keywords:** pancreatic cancer, bioactive compounds, flavonoids

## Abstract

Pancreatic ductal adenocarcinoma (PDA) is a highly challenging malignancy to treat, with a high mortality rate and limited therapeutic options. Despite advances in cancer research, the prognosis for patients diagnosed with PDA is often poor due to late-stage detection and resistance to conventional therapies. Consequently, there is growing interest in the potential of bioactive compounds as alternative or adjuvant treatments, given their ability to target multiple aspects of cancer biology, offering a more holistic approach to treatment. In the context of PDA, certain bioactive compounds, such as polyphenols (found in fruits, vegetables, and tea), flavonoids, carotenoids and compounds in cruciferous vegetables, have shown potential in inhibiting cancer cell growth, reducing inflammation, and promoting cancer cell apoptosis. This review aims to elucidate the mechanisms, by which these bioactive compounds exert their effects, modulating the oxidative stress, influencing inflammatory pathways and regulating cell survival and death. It also highlights current clinical trials that are paving the way toward incorporating these natural agents into mainstream treatment strategies, with the goal of boosting the efficacy of conventional therapies for PDA.

## 1. Introduction

Cancer universally represents one of the largest public health concerns, substantially contributing to global disease burden and mortality, with complex etiological factors including genetic predisposition, environmental exposures, and lifestyle choices contributing to its onset and progression [1,2]. Among lifestyle factors, diet has emerged as a crucial modifiable risk factor that can influence cancer development and progression [2].

Bioactive compounds are extra nutritional constituents that typically occur in small quantities in foods [3], such as vegetables, whole grains, and spices—has garnered significant attention for their potential protective and therapeutic effects against various types of cancer [4]. Bioactive compounds, including polyphenols, flavonoids, carotenoids, and glucosinolates, have been shown to possess a range of anticancer properties, such as antioxidant activity, anti-inflammatory effects, and the ability to modulate key signaling pathways involved in tumorigenesis [4,5,6,7]. These compounds can influence mechanisms such as oxidative stress, cell proliferation, apoptosis, and metastasis, all of which are critical in the development and spread of cancer. Moreover, emerging evidence suggests that bioactive compounds may also enhance the efficacy of conventional cancer treatments [8,9,10,11,12] even against the aggressive pancreatic ductal adenocarcinoma (PDA).

PDA is a devastating disease with a five-year survival rate of about 13% [13]. Resistance to conventional treatment options and the toxicity of current chemotherapy agents, such as gemcitabine, makes PDA a vital target for the development of novel therapeutic agents [14].

This review explores the potential of dietary bioactive compounds in the aggressive context of PDA prevention and therapy, with a focus on their mechanisms of action, their role in modulating cancer-related pathways, and the emerging clinical trials that examine their impact on cancer outcomes.

## 2. Bioactive Agents Against PDA

In the following sections, we will discuss some of the most notable bioactive compounds investigated for their potential roles in preventing and managing pancreatic ductal adenocarcinoma. By examining their mechanisms of action and their contributions to mainstream treatment approaches, we aim to provide a comprehensive overview of how these natural agents might enhance therapeutic strategies against this highly aggressive malignancy.

**Figure 1:**
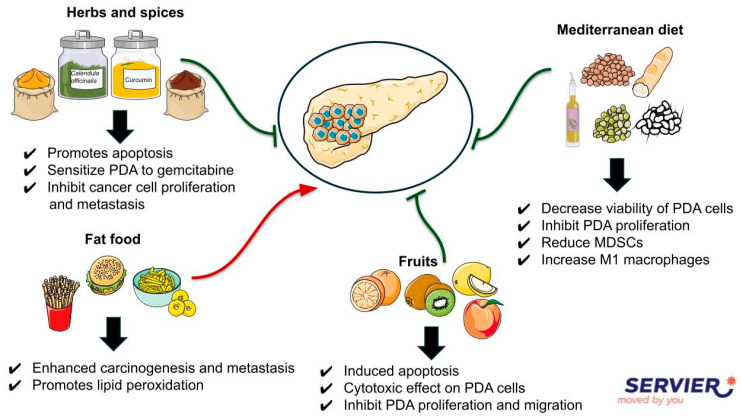
Mechanisms of action of PDA-targeting bioactive compounds. The figure summarizes the main biological effects of the bioactive compounds, highlighting key molecular targets and signaling pathways involved.

As illustrated in Figure 1, the following paragraph will describe the main bioactive compounds and their biological mechanisms.

### 2.1. Olive Oil

The mediterranean diet is primarily a plant-based dietary pattern, consisting of a high intake of fruit, vegetables, legumes, nuts and seeds, whole grains, spices, herbs, and olive oil [15]. Owing to its food composition, the mediterranean diet is a dietary pattern rich in protective nutrients and bioactive compounds able to prevent several diseases, including obesity and cancer [16]. One of the major differences between mediterranean diets and other healthy diets is the high intake of olives and olive oil; the annual intake of olive oil in Mediterranean countries can range from 15.3 to 23 kg per capita [17,18]. Many of the health benefits associated with consuming olive oil have been attributed to its high concentration of biophenols [18]. Adherence to a Mediterranean diet is associated with a reduced risk for heart disease and most cancers, including PDA [19,20].

A recent meta-analysis pooling data from one case–control and three cohort studies found that mediterranean diet was not statistically significantly associated with PDA risk [21]. However, findings from two more recent prospective studies observed reductions for high mediterranean diet adherence of between 18% and 43% in the risk of PDA [22]. Pooled data from observational studies support that high olive oil consumption may protect against upper aerodigestive (composed of oral cavity, pharynx, and larynx) and total gastrointestinal and esophageal subtype cancer risk, but not against colorectal and gastric cancers risk [15,23]. An Italian case–control study showed an inverse relationship between olive oil and PDA [24]. Additionally, oleocanthal-rich extra virgin olive oils can induce lysosomal membrane permeabilization in breast and prostate cancer cells, leading to cellular toxicity [25]. In mouse models engineered to develop pancreatic neuroendocrine tumors, oleocanthal treatment reduced tumor burden and extended lifespan [25].

Studies have shown that olive biophenols, such as oleuropein and hydroxytyrosol (HT), can reduce the viability of PDA cells in vitro [26,27]. These compounds induced morphological changes and caused G2/M cell cycle arrest in PDA cells, suggesting potential therapeutic effects [27]. HT, a compound derived from olive oil, has been shown to have various health benefits, though its therapeutic effect on PDA remains debated. HT inhibited the proliferation of Panc02 cells through the STAT3/Cyclin D1 signaling pathway and in HT-treated tumor-bearing mice, orthotopic PDA tumors were suppressed, with a reduction in myeloid-derived suppressor cells (MDSCs) and an increase in M1 macrophages [28]. Additionally, HT suppressed the expression of immunosuppressive molecules in bone marrow-derived MDSCs, down-regulating C/EBPβ and the phosphorylation of STAT3 [28]. Furthermore, HT enhanced the anti-tumor effects of an anti-CD47 antibody in vivo.

These findings collectively suggest that olive oil and its bioactive compounds may offer potential benefits in the context of PDA. However, further clinical trials are necessary to fully understand their efficacy and therapeutic potential.

### 2.2. Vitamin E Tocotrienols

One of the most compelling groups of anti-tumor bioactive compounds in cereal grains are vitamin E tocotrienols [29]. Tocotrienols are unsaturated, naturally occurring vitamin E compounds, which exist as four isoforms: α-, β-, δ-, and γ-tocotrienol [30]. In PDA, vitamin E δ-tocotrienol (VEDT) is the most potent anticancer agent among the four isomers, both in vitro and in vivo [31]. It was shown that oral administration of 100 mg/kg/day of VEDT to mice resulted in satisfactory bioavailability in mouse pancreas tissue with no significant toxicity [32]. Of note, VEDT administered for almost 1 year, prolonged the survival and delayed pancreatic intraepithelial neoplasia lesions in the LSL-KRASG12D/PDX-1-Cre genetic mouse model of PDA [33].

### 2.3. Marigold Supercritical Fluid Extract

The use of supercritical fluids in green technology, with a particular focus on supercritical CO_2_ in the extraction of compounds with low polarity, can be enhanced by the incorporation of distinct co-solvents to optimize extraction performance.

The antitumoral properties and mechanism of action of a supercritical CO_2_ extract from *Calendula officinalis*, more commonly known as marigold, in the context of PDA, have been investigated [34]. It was observed that marigold supercritical fluid extract diminished the PDA cell viability in a dose dependent manner inducing apoptotic cell death, increasing the percentage of necrotic cells, inhibiting the anchorage-independent cell growth, and synergizing with the chemotherapeutic drug 5-fluorouracil, used in clinics [35,36,37]. Additionally, marigold supercritical fluid extract stimulates the expression of BMP8B, causing an energetic imbalance that ultimately results in autophagy-induced cell death [34]. Given that metabolic reprogramming is a well-known hallmark of cancer, the direct influence of marigold extract on PDA cell metabolism warrants further investigation into its potential as co-adjuvant in patient therapy.

Another study highlights that marigold extracts may also protect pancreatic β-cells from oxidative damage induced by arsenic exposure [38]. This protective effect is mediated through the activation of the Nrf2 signaling pathway, highlighting the potential of marigold extracts in preserving pancreatic cell function [38].

While preclinical studies have demonstrated that marigold extracts possess antitumoral properties against PDA cells [34,35,36,37], there is a lack of clinical trials directly evaluating marigold extract as a treatment for PDA patients.

### 2.4. Polyunsaturate Fatty Acids

Epidemiological studies suggest a correlation between dietary fat intake and carcinogenesis, with several authors proposing that certain polyunsaturated fatty acids (PUFAs) may have modulatory effects [39,40,41,42,43]. Although n-3 fatty acids (like eicosapentanoic acid (EPA) and docosahexaenoic acid (DHA)) are supposed to suppress tumor proliferation, n-6 PUFA (e.g., linoleic acid) enhanced carcinogenesis and metastasis in various trials [44]. While the beneficial effects of PUFAs remain a topic of debate in the literature, several studies highlight their antioxidant properties, suggesting a role in enhancing cellular defense mechanisms and inhibiting the arachidonic acid cascade [45]. Conversely, other studies propose that the tumor-suppressive effects of n-3 PUFAs may result from their preferential accumulation in tumor cells. This selective enrichment may render tumor cells more susceptible to oxidative stress and lipid peroxidation, thereby inducing cytotoxic effects and contributing to the inhibition of tumor growth [46,47,48]. In addition, Gregor and colleagues demonstrated that lipid peroxidation was increased in tumor-free pancreas tissue of a standard high fat diet rich in n-6 PUFA-10 mg N-nitrosobis-2-oxypropylamine, compared to the high fat diet rich in n-6 PUFA, the diet rich in n-3 PUFA—and a diet containing a mixture of n-3, n-6 and n-9 PUFA, which might be explained by the fact that n-6 PUFA are prone to lipid peroxidation initiated by reactive oxygen species and may promote carcinogenesis and metastasis [45]. In contrast to previous studies [46,49,50], the level of lipid peroxidation was decreased in intratumoral tissue compared to tumor-free pancreas [45].

### 2.5. Citrus Fruits

The role of fruits and vegetables consumption on pancreatic cancer risk has not been established yet, as most of the studies on the topic reported non-significant results [51].

Citrus, the genus *Citrus L.* of the family *Rutaceae*, subfamily *Aurantioideae* [52], is one of the most important fruit crops, including pomelo, sweet orange, sour, lemon, lime, citron, grapefruit, kumquat, and hybrids are rich in phytonutrients, offering various health benefits [53,54,55]. They are a major source of flavonoids, such as hesperidin, narirutin, and rutin, which have antioxidant, anti-inflammatory, and anticancer properties [56]. Citrus fruits are also high in carotenoids like β-carotene, lutein, and lycopene, which may reduce the risk of cardiovascular disease, macular degeneration, and cancer [57]. Additionally, citrus limonoids, including limonin and nomilin, are known for their antioxidant, anticancer, and hypocholesterolemic effects [58].

A plethora of epidemiological studies, encompassing case–control and cohort designs, have been conducted to investigate the association between the consumption of citrus fruits and the risk of developing PDA [59,60,61,62,63,64,65,66,67,68,69]. A number of these studies have indicated an inverse relationship between the intake of citrus fruits and the likelihood of contracting the aforementioned disease [59,61,62,66,67,68,69]. Of note, when total fruit intake was divided into citrus fruit or yellow-orange fruit, the intake of yellow-orange fruit was positively associated with pancreatic cancer risk among normal-weight participants [64]. Larsson et al. emphasize the potential overstatement of relationships observed in case–control studies, due to the assessment of dietary intake occurring subsequent to the diagnosis of pancreatic cancer, a process which may result in the occurrence of recall bias. Furthermore, selection bias is a problem in situations of low participation rates among controls because those who participate are likely to be more health conscious and, therefore, are likely to consume more fruits and vegetables than nonrespondents. The potential for selection bias may be introduced if the case series is restricted to cases in which subjects are still alive at the time of interview. Case–control studies of pancreatic cancer are particularly vulnerable to bias, owing to the elevated and expeditious fatality rates associated with the condition [60]. In addition, Silverman et al. demonstrate, a statistically significant correlation between obesity and a 50–60% increased risk of PDA, which remains consistent across both sex and race demographics [63]. No important associations were observed with citrus fruit and juice consumption in a prospective study on United States adults with no reported history of cancer [65].

Citrus bioactive compounds have the ability to inhibit multiple stages of breast [70], colon [71], prostate [72], lung [73] and PDA [74,75]. Lime juice extracts inhibited cancer cell growth in a dose-dependent manner, with the methanol extract showing the highest activity [74]. Protein-level analysis for p53, Bax, Bcl-2, and caspase-3 indicated that the extracts promoted apoptosis in the cancer cells [76]. Additionally, limonoids—such as limonin glucoside, limonexic acid, isolimonexic acid, and limonin—extracted from lime seeds also inhibited PDA cells through apoptosis [75].

### 2.6. Flavonoids

Flavonoids are bioactive compounds found in various sources, such as citrus fruit, apples, green tea, berries, and grapes [77,78]. Consumption of flavonoid-rich foods has been linked to a reduced risk of several diseases, including obesity, cancer, and heart disease [79]. Flavonoids are the most common of the plant polyphenolics [80] and are thought to have chemoprotective properties [81].

Based on the degree of substitution, flavonoids are further subdivided into chalcones, flavanones, flavones, flavonols, flavanols, isoflavones, and anthocyanins [82]. Moreover, studies reveal that flavonoids, such as isoliquiritigenin [83], apigenin [84], quercetin [85], among others exert significant anticancer effects in various cancers [86].

Flavones have shown inhibition of PDA cell growth in vitro [87], and quercetin (a flavonol) has demonstrated inhibition of PDA growth and prevention of metastasis in vitro and in vivo [88].

Epidemiological studies on flavonoids and PDA suggest an inverse association with intake of specific flavonoids, but results are inconsistent and based on few cases [89,90,91].

#### 2.6.1. Broussoflavonol B

Broussoflavonol B (Bf-B), a flavonoid compound identified in the roots of Daphne giraldii Nitsche, has been extensively investigated for its potential anti-inflammatory, antioxidant, and anticancer properties [92]. The results demonstrated that Bf-B with diisopentenyl has potent cytotoxic effects on PANC-1 cancer cells. AURKA, PLK1, and MET might serve as key targets for Bf-B inhibition of disease progression in PDA patients [92]. The results demonstrated that Bf-B inhibits the proliferation and migration of PANC-1 and BXPC-3 cells and induces cell cycle S-phase arrest, apoptosis, and DNA damage [92].

#### 2.6.2. Isorhamnetin

Isorhamnetin (ISO), 3′-methylquercetin, is a dietary flavonoid found in numerous plants such as red onion, broccoli, gingko biloba leaves, sea buckthorns, apples, pears, green grapes [93]. ISO showed anti-proliferative effect on several types of cancer cells such as skin, colon, breast, and PDA cells by inducing apoptosis, inhibiting proliferation, modulating signaling pathways, and exerting antioxidant effect [94,95,96,97]. Recently, it was also shown that the cytostatic effect of ISO on human cancer-associated-fibroblast (CAFs) impacts the tumor growth and development of chemoresistance. In particular, Ganbold and colleagues, demonstrated that in PDA-derived CAFs, ISO induce cell cycle arrest at G2/M phase associated with activation of p21, impaired mitochondrial homeostasis, and inhibition of inflammatory mediators gene expression [93]. In addition, it was shown in a PDA xenograft mouse model, that the combined administration of gemcitabine and flavopiridol demonstrated a significant reduction in tumor volume and induction of apoptosis [98].

#### 2.6.3. Apiin, Rhoifolin and Vitexin

Apiin, rhoifolin, and vitexin are flavonoid glycosides found in various plants, each with distinct bioactive properties that contribute to their potential health benefits. Apiin, primarily found in celery and parsley, has shown anti-inflammatory, antioxidant, and anticancer activities, and may also help in regulating blood pressure and supporting cardiovascular health [99]. Rhoifolin, typically present in citrus fruits, has demonstrated anticancer, anti-inflammatory, and antioxidant effects, with studies suggesting it inhibits tumor cell proliferation and metastasis while modulating key signaling pathways like NF-κB and MAPK [100]. Vitexin, found in plants such as passionflower and hawthorn, is known for its anti-inflammatory, antioxidant, and neuroprotective properties [101].

Cell viability assay revealed that apiin, rhoifolin, and vitexin could inhibit proliferation of PDA cell lines, with rhoifolin showing the maximum inhibitory effect [102]. Rhoifolin inhibited cell proliferation and promoted apoptosis of PDA cells, which was associated with up-regulated JNK and p-JNK as well as down-regulated p-AKT [102]. Rhoifolin also inhibited cell migration and invasion and increased the antioxidant capacity in PANC-1 and ASPC-1. In addition, AKT activator or JNK inhibitor effectively reversed the anticancer effects of rhoifolin in PDA [102].

#### 2.6.4. Hispidulin

Hispidulin (4′, 5, 7-trihydroxy-6-methoxyflavone) is one of the most studied flavonoids, primarily present in plants of the *Asteraceae* [103,104,105] and *Lamiaceae families* [106]. Hispidulin has a wide range of biological activities, including anti-inflammatory, antifungal, antiplatelet, anticonvulsant, antiosteoporotic, and notably anticancer activities [107]. Moreover, hispidulin exhibits synergistic anti-tumor effects when combined with some common clinical anticancer drugs. Indeed, hispidulin enhances the chemosensitivity of bladder cancer cells to gemcitabine and 5-Fluorouracil by suppressing the HIF-1α/P-gp signaling cascade [108], sensitizes renal cell carcinoma cells to sunitinib-induced growth suppression, G0/G1 arrest, and apoptosis by regulating the Stat3 pathway [109], enhances the anti-tumor activity of temozolomide by promoting ROS generation and regulating the AMPK/mTOR signaling pathway in glioblastoma [110] and sensitizes SKOV3 cells (human ovarian cancer cells) to TRAIL-induced apoptosis and converts TRAIL-resistant cells to TRAIL-sensitive cells [111]. The combination of hispidulin and chemotherapeutic drugs reduces the efflux of chemotherapeutic drugs, enhances the chemosensitivity of cancer cells, and reverses drug resistance [112]. In a human PDA mouse xenograft model, oral administration of hispidulin has been shown to suppress tumor growth and angiogenesis, without significant toxicity [112]. Similarly, in vitro results indicate that endothelial cells are more sensitive to hispidulin compared to PDA cells, and hispidulin inhibits VEGF-induced cell migration and tubular formation in endothelial cells [112].

#### 2.6.5. Isoorientin

Isoorientin is a 6-C-glycosylflavone, present in many plant species, such as corn (*Zea mays*) silks and pollens, kudzu (*Pueraria tuberosa*), *Patrinia villosa* [113]. Isoorientin exhibits antioxidant, antiviral, analgesic, antitumor, and anti-inflammatory activities [114,115,116]. In PDA, isoorientin significantly inhibited cell survival, induced apoptosis, and reduced malignancy by reversing epithelial–mesenchymal transition, matrix metalloproteinase expression, and decreasing vascular endothelial growth factor levels [117]. Furthermore, the AMP-activated protein kinase (AMPK) signaling pathway was strongly activated by isoorientin treatment [117]. However, in PDA cells transfected with a lentivirus to interfere with the expression of the PRKAA1 (protein kinase AMP-activated catalytic subunit alpha 1) gene, there were no significant differences in apoptosis rates or malignancy biomarker expression between the isoorientin-treated and untreated groups [117].

#### 2.6.6. Naringenin

Naringenin ((2S)-5,7-dihydroxy-2-(4-hydroxyphenyl)-2,3-dihydrochromen-4-one) is a flavanone, a type of flavonoid, and is colorless and odorless [118,119]. Naringenin is the most abundant in grapefruit, yuzu, pummelo, orange, tangerine and lime [120]. Naringenin inhibited PDA by suppressing the TGF-β signaling pathway, a key regulator of epithelial–mesenchymal transition (EMT). It also reduced cell migration through caspase-3 cleavage, elevated reactive oxygen species levels, and induced cell death via apoptosis signal-regulating kinase (ASK)-1. By inhibiting the TGF-β/Smad-3 pathway, naringenin decreased the expression of EMT markers [121]. Naringenin augmented the sensitivity of PANC-1 cells to gemcitabine [121]. Finally, naringenin increased ROS levels in PDA SNU-213 cells and induced ASK-1-mediated cell death [122]. A reduction in the expression of p38, JNK, p58 and peroxiredoxin-1, a regulator of oxidative stress and cell homeostasis, was observed when SNU-213 cells were treated with naringenin [122].

#### 2.6.7. Kaempferol

Kaempferol (KAE), a natural flavonoid widely present in a variety of plant-based foods (i.e., Leafy greens, fruits, cruciferous vegetables, herbs, tea, legumes), with significant anti-tumor and anti-inflammatory properties. Recent studies have explored its ability to sensitize PDA cells and mouse models to Erlotinib [123]. In vitro, the combination of KAE and Erlotinib markedly inhibited cell proliferation and promoted apoptosis, compared to Erlotinib alone. Network pharmacology analysis suggested that KAE enhances Erlotinib’s effect in PDA, potentially through the PI3K/AKT signaling pathway and EGFR TKI resistance mechanisms [123]. Notably, survival analysis revealed that PDA patients with high EGFR expression had lower survival rates. In vivo, the combined treatment of KAE and Erlotinib significantly reduced the volume and weight of subcutaneously grafted tumors [123].

#### 2.6.8. Puerarin

Puerarin is a natural flavonoid extracted from the roots of the kudzu plant or the kudzu vine [124]. Puerarin has various pharmacological effects, such as enhancing the circulatory system function, reducing myocardial oxygen consumption, decreasing blood sugar, and preventing hypertension and arteriosclerosis [125]. Puerarin induced mitochondrial-dependent apoptosis in PDA cell lines by disrupting the balance between Bcl-2 and Bax [124]. It also inhibited PDA cell migration and invasion by counteracting epithelial–mesenchymal transition [124]. In a nude mouse model, puerarin administration reduced PDA growth and metastasis [124]. Mechanistically, puerarin exerted its therapeutic effects by suppressing the Akt/mTOR signaling pathway. Notably, puerarin is bound to the kinase domain of the mTOR protein, altering the activity of surrounding amino acid residues associated with the ATP-Mg2+ complex [124]. Additionally, puerarin impaired glucose uptake and metabolism by decreasing the oxygen consumption rate and extracellular acidification rate, both of which were dependent on HIF-1α and the glucose transporter GLUT1 [124].

#### 2.6.9. Fisetin

Fisetin is a flavonoid that occurs naturally in a variety of plant species and has a wide range of functionalities, including anti-inflammatory, antioxidant and anticancer properties [126,127]. Fisetin treatment was shown to inhibit the growth of chemoresistant PDA cells [128]. It induced apoptosis and suppressed the invasion of AsPC-1 PDA cells by inhibiting DR3-mediated NF-κB activation. cDNA array analysis revealed that fisetin altered the expression of over twenty genes, with the most significant decrease observed in DR3 expression, and a parallel increase in IκBα, the NF-κB inhibitor. Down-regulation of DR3 led to reduced activation of NF-κB/p65, MMP-9, and XIAP, all of which are associated with chemoresistance in PDA cells [128]. Additionally, transient knockdown of DR3 using RNA interference, along with blocking the DR3 receptor with an extracellular domain antibody, significantly enhanced fisetin-induced effects on cell proliferation, invasion, and apoptosis, accompanied by decreased MMP-9, XIAP, and NF-κB DNA binding activity [128].

#### 2.6.10. Wogonin

Wogonin is a flavonoid compound extracted from the root of *Scutellaria baicalensis* [129]. It has antioxidant activity, and antiinflammatory, anti-tumor, immunomodulatory, neuroprotective effects [130]. Wogonin also acts as a chemosensitizer, reducing drugresistance in cancer therapy. When wogonin is used in combination with anticancer drugs such as etoposide, doxorubicin, 5-FU, and cisplatin [131], it can induce tumor cell apoptosis [132] and protect normal cells from side effects. In addition, Xing et al., reported that wogonin enhanced the sensitivity of ovarian cancer cells to gemcitabine by inhibiting the PI3K/Akt signaling pathway [133], while bioinformatics results predicted that wogonin promoted PDA cell apoptosis by inhibiting protein kinase B (Akt) signaling, thereby enhancing the sensitivity of gemcitabine to PDA [134].

It is reported that FV-429, a derivative of the natural flavonoid wogonin, inhibited the invasion and metastasis of PDA cells by modulating Epithelial–mesenchymal transition-related proteins [135]. In addition, FV-429 inhibits migration, invasion, and metastasis of human PDA cells by affecting the Hippo/YAP1 pathway both in vivo and in vitro [135].

#### 2.6.11. Isoliquiritigenin

Isoliquiritigenin (ISL) is a bioactive flavonoid isolated from licorice, the ground root of *Glycyrrhiza glabra* [136]. ISL exhibits numerous pharmacological properties, such as anti-inflammatory, anti-microbial, antioxidative, anticancer as well as immunoregulatory effects [137]. Zhang and colleagues showed that ISL inhibited PDA cell growth and induced apoptosis, both in vitro and in vivo [136]. ISL caused accumulation of autophagosome through blockade of late stage autophagic flux [136]. Of note, ISL synergistically sensitized the cytotoxic effect of gemcitabine and 5-fluorouracil on PDA cells as both drugs induced autophagy. Molecular docking analysis has indicated that ISL acted by direct targeting of p38 MAPK, which was confirmed by ISL-induced phosphorylation of p38. The autophagy flux induced by p38 inhibitor SB203580 was blocked by ISL, with further increasing toxicity of ISL in PDA cells [136].

#### 2.6.12. Luteolin

Luteolin (Lut), is a flavonoid, specifically a flavone, found in celery, green pepper, parsley, and perilla leaf. Lut suppressed pancreatocarcinogenesis and reduced the expression of dihydropyrimidine dehydrogenase (DPYD), an enzyme that degrades pyrimidines such as 5*-*fluorouracil, in PDA [138]. Lut exhibits strong antioxidant activity and shows antiinflammatory and antitumor effects against different tumor among which PDA [138,139,140,141,142,143]. Recently, it was observed the therapeutic effects of the combined treatment with 5*-*FU and Lut in PDA resulted in remarkable therapeutic effects both in vitro and in vivo, whereas 5*-*FU or Lut alone showed no significant effects [138].

#### 2.6.13. Anthocyanins

Anthocyanins, found in various pigmented plants as secondary metabolites, represent a class of dietary polyphenols known for their bioactive properties, demonstrating health-promoting effects against several chronic diseases [144]. Cyanidin-3-O-glucoside (C3G) is a major anthocyanin found in various fruits, particularly berries, and is known for its antioxidant and anti-inflammatory properties. Upon ingestion, C3G undergoes metabolism in the human body, where it is hydrolyzed by intestinal enzymes into its aglycone form, cyanidin, which is further absorbed and distributed [145,146]. The bioavailability of C3G is relatively low, but its metabolites may still exert beneficial effects on various cellular processes, including reducing oxidative stress and modulating inflammatory pathways. Notably, studies have shown that C3G can induce apoptosis in colon cancer and glioblastoma cells by modulating oxidative stress pathways [147]. Additionally, research indicates that C3G exerts protective effects on pancreatic beta cells by alleviating palmitic acid-induced dysfunction through the regulation of endoplasmic reticulum stress pathways [147].

Kuntz et al., showed that 60 min after a single anthocyanins dose, plasma extracts from volunteers inhibited migration of the PDA cell line PANC-1 in vitro [148]. In a later study, the same group showed that plasma extracts had different effects on cancer cell migration in vitro depending on the cancer cell line [149]. In PANC-1, extracted-plasma metabolites after the administration of anthocyanins-rich juice reduced cell migration significantly in comparison to plasma extracts after placebo. By contrast, no reduction was observed for the migration of AsPC-1. The observed discrepancy was attributed to the diminished migration and expression of cell adhesion molecules in PANC-1 cancer cells in vitro, as evidenced by the activation of FAK- and NF-kB-pathways, along with the reduction in ROS [149].

Anthocyanins exert antitumor effects through multiple mechanisms. In the early stages, they inhibit inflammation and prevent normal cell transformation by regulating antioxidant enzymes. During carcinogenesis, they target key signaling pathways like MAPK and AP-1, inhibiting RTK activity and causing cell cycle arrest and DNA repair [150]. In later stages, anthocyanins promote cancer cell apoptosis by activating caspases and reduce metastasis by targeting VEGF signaling. Additionally, they help overcome multidrug resistance, improving chemotherapy sensitivity. These actions are mediated through several molecular pathways, including Ras-MAPK, PI3K/Akt, and NF-κB [150].

While anthocyanins has demonstrated anticancer properties in preclinical studies [149,150,151], there is a notable absence of clinical trials specifically investigating its efficacy in treating PDA. Further research is necessary to fully understand the therapeutic potential and mechanisms of anthocyanins in PDA prevention and treatment.

#### 2.6.14. Xanthohumol, Resveratrol, Phenethyl Isothiocyanate, Indole-3-Carbinol

Xanthohumol, resveratrol, phenethyl isothiocyanate (PEITC), and indole-3-carbinol are bioactive compounds with promising health benefits, particularly in cancer prevention and treatment.

Xanthohumol (XN) is a prenylated flavonoid compound primarily found in hops (*Humulus lupulus*), the key ingredient used in beer production. Xanthohumol exhibits antioxidant, anti-inflammatory, and anticancer properties by modulating various cellular pathways involved in tumor growth and metastasis [152,153]. The regulation by xanthohumol of the Nrf2/NF-kB/mTOR/AKT pathways induce a strong antioxidant and anti-inflammatory effect, among others the acceleration of autophagy through increased synthesis of Bcl-2 proteins, inhibition of the synthesis of VEGF responsible for angiogenesis and phosphorylation of Hexokinase II [152].

Resveratrol, a polyphenol found in grapes, berries, and red wine, is known for its antioxidant, anti-inflammatory, and anticancer effects, including its ability to inhibit tumor cell proliferation and induce apoptosis [154,155]. Notably, it was observed that resveratrol might be involved in regulating EMT in the PDA microenvironment [156].

PEITC, derived from cruciferous vegetables like watercress, is a potent anticancer agent, known for its ability to induce cancer cell death and inhibit metastasis through various molecular mechanisms [157,158]. PEITC targets crucial cellular signaling pathways involved in cancer progression, notably the NF-κB, Akt, and MAPK pathways [159]. Regarding the studies with animal models, Stan et al. demonstrated that oral administration of PEITC reduced pancreatic cancer cell growth in a MIAPaca2 xenograft animal model of 6 weeks old [160].

Indole-3-carbinol, also found in cruciferous vegetables such as broccoli and cabbage, has demonstrated anticancer effects by regulating estrogen metabolism, modulating signaling pathways involved in cell growth, and promoting apoptosis in cancer cells [157]. Interestingly, the mixture of xanthohumol and PEITC was found to be the most potent modulator of the Nrf2 pathway in human PDA cell line [161].

Together, these compounds contribute to cancer prevention and therapy, with ongoing research exploring their combined therapeutic potential.

### 2.7. Chinese Herbs

Chinese herbs have long been utilized in traditional medicine for their potential therapeutic properties, particularly in the treatment of cancer. Several studies have highlighted the bioactive compounds derived from Chinese herbs that show promise in PDA treatment. For instance, compounds such as curcumin, found in *Curcuma longa* (turmeric), and berberine, isolated from *Coptis chinensis*, have been shown to exhibit anticancer properties by modulating various cellular pathways involved in cancer cell proliferation, apoptosis, and metastasis [162,163,164,165]. Additionally, the active ingredients in *Glycyrrhiza uralensis* (licorice) have demonstrated anti-inflammatory and anti-tumor activities, which may aid in reducing the aggressiveness of PDA [166]. Recent research underscores the potential of these herbs as adjuncts to conventional therapies, aiming to enhance the effectiveness of treatment while minimizing side effects [167]. However, more clinical trials are necessary to validate these findings and determine their safety and efficacy in PDA treatment.

#### 2.7.1. Curcumin

Curcumin, a component of turmeric (*Curcuma longa*), is one such agent that has been shown to suppress the transcription factor nuclear factor-κB (NF-κB), which is implicated in proliferation, survival, angiogenesis, and chemoresistance [168]. It was shown that curcumin can sensitize PDA to gemcitabine in vitro and in vivo. In vitro, curcumin inhibited the proliferation of various PDA cell lines, potentiated the apoptosis induced by gemcitabine, and inhibited constitutive NF-κB activation in the cells [168]. In vivo, tumors from nude mice injected with PDA cells and treated with a combination of curcumin and gemcitabine showed significant reductions in volume, Ki-67 proliferation index, NF-κB activation, and expression of NF-κB–regulated gene products (cyclin D1, c-myc, Bcl-2, Bcl-xL, cellular inhibitor of apoptosis protein-1, cyclooxygenase-2, matrix metalloproteinase, and vascular endothelial growth factor) compared with tumors from control mice treated with olive oil only [168]. The combination treatment was also highly effective in suppressing angiogenesis [168].

#### 2.7.2. Thymoquinone (From *Nigella sativa* Seeds)

Thymoquinone is the principal bioactive compound obtained from the seeds of *Nigella sativa*, a plant commonly referred to as black cumin. Traditional remedies have utilized these seeds for centuries, and modern research has validated thymoquinone’s anti-inflammatory, antioxidant, and proapoptotic properties in a range of malignancies. In PDA, it has been shown to suppress cell growth and survival by modulating pivotal pathways such as NF-κB and PI3K/Akt, both of which play essential roles in tumor proliferation and angiogenesis [169,170]. Notably, this compound also appears to heighten the efficacy of conventional chemotherapeutics, including gemcitabine and oxaliplatin, possibly by enhancing cancer cell sensitivity to apoptosis [171,172,173]. Researchers suggest that its capacity to overcome drug resistance stems from the downregulation of multiple survival pathways [174]. While most findings to date are based on in vitro and in vivo preclinical models, the evidence strongly supports the potential of thymoquinone as part of a combination strategy against PDA [175]. Further investigation into its clinical application and synergistic effects with standard therapies is warranted to define optimal treatment protocols.

#### 2.7.3. Alpinumisoflavone

Alpinumisoflavone (AIF) is a prenylated isoflavone originated in *Cudrania tricuspidate* with versatile bioactive properties, including anticancer activity. As it is widely spread in East Asia and known for its versatile bioactive properties, it was utilized for a medical herb in traditional Chinese medicine [176]. Different studies showed that AIF suppressed cell proliferation, migration, and invasion capacity of tumoral cells [177], favor apoptosis [178] and suppress the tumor growth and metastatization [177,179,180]. As a result, AIF has garnered significant attention as a potential chemotherapeutic adjuvant [181]. Recent in silico studies have suggested AIF’s capability to target receptors associated with the angiogenesis pathway [182]. In addition, different studies have demonstrated that combining AIF with standard anticancer drugs enhances its therapeutic efficacy against several cancers including PDA [183,176]. In fact, AIF: (i) suppressed PDA cell viability, (ii) disrupted the normal formation of PANC-1 and MIAPaca2 spheroids in vitro model, (iii) weakened cell migratory ability by downregulating mesenchymal proteins, (iv) strengthened apoptosis induction, oxidative stress, mitochondrial calcium dysregulation, depolarization, and (v) OXPHOS impairment in PDA cells [176]. Of note, the combination of AIF and gemcitabine synergistically induced mitochondrial dysfunction in PDA cells [176].

#### 2.7.4. Piperlongumine

Piperlongumine is an alkaloid extracted from long pepper, a staple in various traditional Asian medical practices. One of its distinguishing features is the selective increase in oxidative stress in cancer cells, which in turn triggers apoptosis without major harm to normal cells [184]. Studies focused on PDA indicate that piperlongumine can curtail tumor growth and migration by elevating intracellular reactive oxygen species and suppressing pro-survival pathways [185]. In addition, it disrupts NF-κB signaling, reducing the expression of genes essential for tumor progression [186]. Several preclinical investigations point to its potential synergy with gemcitabine, as enhanced oxidative stress can sensitize PDA cells to chemotherapy [187]. Transcriptome analyses further reveal that the compound engages both oxidative and endoplasmic reticulum stress mechanisms, providing a multifaceted assault on tumor cells [187]. Although clinical data are still lacking, these promising laboratory findings suggest that piperlongumine may hold significant therapeutic value, especially in combination with existing standard-of-care treatments.

#### 2.7.5. Honokiol

Honokiol is a polyphenol derived from the bark of Magnolia officinalis, traditionally prized in Chinese medicine for its broad-ranging benefits, including antioxidant and anti-inflammatory effects. Research on PDA has shown that honokiol interferes with oncogenic signaling networks—namely STAT3 and NF-κB—thereby inhibiting tumor cell proliferation and enhancing apoptosis [188]. It also appears to impede the complex interplay between tumor and stromal cells, a critical factor in PDA progression and metastasis [189]. Notably, combining honokiol with conventional chemotherapy, such as gemcitabine, has led to improved anticancer outcomes in preclinical models, partly due to its capacity to block multiple pathways linked to resistance [188]. Additional findings suggest that honokiol exerts an antiangiogenic effect, limiting the formation of new blood vessels, and thus restraining tumor growth [190]. Although these data remain largely at the laboratory stage, the compound’s ability to target diverse cancer-promoting mechanisms underscores its potential for integration into future therapeutic regimens for PDA malignancies.

## 3. Active Clinical Trials with Bioactive Compounds for PDA

The favorable outcomes observed in both in vitro and in vivo experiments employing bioactive compounds in the treatment of PDA have prompted clinicians and oncologists to investigate the potential of these dietary supplements to enhance postoperative recovery (Table 1). The aim of these study is to examine the effect of dietary prescription with and without nutrition supplementation in PDA patients. In fact, an accelerated recovery may improve outcomes after surgery following complex abdominal operations resulting in a shorter length of stay in PDA patients. It may also help patients to mobilize more quickly and return to the home setting, decrease hospital-acquired infectious complications, and increase potential cost savings. In Table 2, the effectiveness of conventional chemotherapy regimens and dietary supplement is shown. Given the aggressive nature and poor prognosis of PDA, investigating the combined effectiveness of conventional chemotherapy regimens and dietary supplements offers a promising strategy to enhance therapeutic outcomes, overcome chemoresistance, and improve patient quality of life.

Clinical studies have demonstrated that both diet and exercise significantly impact recovery and the effectiveness of chemotherapy in PDA patients. Rosebrock and colleagues found that exercise is feasible and safe for these patients, leading to improvements in quality of life, reduction in cancer-related fatigue, and increased muscle strength [193]. Similarly, research indicates that regular physical activity can help manage side effects of chemotherapy, such as fatigue, and may enhance the efficacy of treatment. Moreover, a balanced diet rich in fruits, vegetables, lean proteins, and whole grains supports nutritional status, reduces the risk of malnutrition, and may alleviate chemotherapy side effects like nausea and fatigue [194].

Incorporating exercise into daily routines has also been associated with improved mental health, quality of life, and reduced anxiety and depression among cancer patients. Additionally, studies suggest that regular exercise and a balanced diet can positively influence survival rates, underscoring the importance of these interventions during cancer treatment [195].

Integrating appropriate diet and exercise regimens into the care plan of PDA patients undergoing chemotherapy can lead to improved recovery, enhanced treatment efficacy, and better overall outcomes. However, it is essential to tailor these interventions to individual patient needs and consult healthcare professionals before making significant changes to diet or physical activity.

## 4. Discussion

PDA is one of the most aggressive cancers because it often remains undetected until advanced stages, shows a dense fibrotic tissue around the tumor cells (desmoplasia), and develops resistance to common treatments. In recent years, many studies have explored how natural bioactive compounds, found in fruits, vegetables, spices, and medicinal plants, might improve treatment results for this type of cancer. These substances include polyphenols (for example, curcumin or resveratrol), flavonoids (such as quercetin, apigenin, and hispidulin), tocotrienols (especially δ-tocotrienol), and certain isothiocyanates. Research shows that they can interfere with multiple signaling pathways in cancer cells, like NF-κB and STAT3 (which promote inflammation and cell survival), PI3K/Akt (involved in cell growth), and processes that lead to tumor invasion (like the epithelial–mesenchymal transition). By working on several targets at once, these molecules might prevent the tumor from quickly becoming resistant, a frequent problem when a therapy only blocks a single pathway.

A key advantage of these bioactive compounds is how they sometimes boost the effectiveness of chemotherapy. For instance, curcumin can strengthen gemcitabine’s impact by lowering the levels of proteins that help cancer cells avoid death (like Bcl-2) and reducing NF-κB activity [196]. Flavonoids like quercetin and kaempferol may also help gemcitabine work better, mainly by increasing the oxidative stress in cancer cells and blocking proteins that make them drug-resistant [197]. Moreover, some of these substances seem to modify the tumor’s microenvironment, reducing the presence of immunosuppressive cells (e.g., myeloid-derived suppressor cells) and breaking the harmful exchange of signals between the tumor and the surrounding tissue [198,199].

Even with these encouraging discoveries, it is not simple to bring these findings into the clinic. Many of these compounds have poor oral bioavailability, meaning only a small portion is absorbed when taken by mouth. This has led to experiments with new techniques, like nano-encapsulation, to deliver them more effectively to the tumor [200,201,202]. Additionally, the different extraction methods from natural sources can cause variability in the final purity of these products, making it difficult to compare studies or reproduce results [203,204]. Although some clinical trials focus on combining nutritional supplements (like fish oil high in n-3 fatty acids or specific high-protein formulas) with standard anticancer treatments, more robust investigations are needed to see if these truly improve survival and quality of life [205].

Finally, recent attention has turned to combining these natural compounds with healthy diets and exercise programs. Physical activity and balanced nutrition are known to reduce overall inflammation and enhance general health, possibly adding to the beneficial effects of bioactive agents. In the future, using these strategies together with chemotherapy, dietary supplements, and lifestyle changes, could improve outcomes for patients with this highly resilient cancer. To reach this goal, well-designed clinical trials will be essential for defining the best doses, the most effective combinations, and the ideal schedules for administration, ensuring safer and more successful therapies.

## 5. Conclusions

Despite the limited therapeutic options and poor prognosis associated with PDA, the integration of bioactive compounds into current treatment strategies represents a promising area of research. Preclinical studies have demonstrated that various natural compounds—such as polyphenols, isothiocyanates, and omega-3 fatty acids—can modulate key molecular pathways involved in tumor growth, inflammation, and chemoresistance. While these findings provide a strong rationale for the adjunctive use of bioactive compounds alongside conventional chemotherapy, clinical evidence remains limited and often inconclusive. Rigorous, well-designed clinical trials are urgently needed to validate their efficacy, determine optimal dosing regimens, and assess potential interactions with standard treatments. Advancing this line of research may pave the way for more effective, integrative therapeutic approaches in pancreatic cancer care.

## Figures and Tables

**Table 1:** Clinical trial with dietary supplement in PDA treatment.

NCT	Therapy	Sponsor
NCT02681601	Omega rich fish oil supplement	Jonsson Comprehensive Cancer Center
NCT02517268	Liquid or solid post operative diet	Sidney Kimmel Cancer Center at Thomas Jefferson University [191]
NCT03187028	Diet alone vs. diet+exercise	University of Alabama at Birmingham
NCT06833658	Aromatherapy with essential oil intervention	Peking University First Hospital
NCT06852014	Peptamen 1.6 supplement	Fundación Pública Andaluza para la Investigación de Málaga en Biomedicina y Salud
NCT03244683	Oral Nutritional Supplementation combined with resistance training	Ohio State University
NCT02940067	Components of the mediterranean diet and exercise training	Royal Surrey County Hospital NHS Foundation Trust
NCT04306874	High-protein nutritional supplementation	Thomas Jefferson University
NCT03167814	No long-chain triglycerides	Helsinki University Central Hospital
NCT06069297	Exercise training, nutritional therapy and anxiety reducing techniques	IRCCS San Raffaele

**Table 2:** Clinical trial with dietary supplement associated with conventional chemotherapy PDA therapy.

NCT	Therapy	Sponsor
NCT06090916	Conventional vs. Dietary and physical activity using MyFitnessPal smartphone app	Jonsson Comprehensive Cancer Center
NCT06050395	Anti-inflammatory and pro-inflammatory dietary patterns	H. Lee Moffitt Cancer Center and Research Institute
NCT06595160	Plant-based diet	Emory University
NCT06149546	High protein, high energy diet, Fish oil supplement, Pancreatic Enzymes	Cancer Trials Ireland
NCT05420259	Exercise and Dietary Intervention	Hospital Beatriz Ângelo
NCT04837118	Dietary intervention	M.D. Anderson Cancer Center
NCT04188990	Dietary advice, Oral Nutritional Supplementation, Enteral Feeding or Parenteral Nutrition	Hospital Galdakao-Usansolo
NCT02336087	Curcumin, vitamin D, vitamin K2, vitamin K1, B-6, high selenium broccoli sprouts, epigallocatechin gallate, L-carnitine, garlic extract, genistein, zinc amino chelate, mixed toxopherols, ascorbic acid, D-limonene	City of Hope Medical Center
NCT03958019	Supervised and self-managed exercise, dietary counseling, and education sessions	University of Dublin, Trinity College [192]
NCT06412510	30 gm protein supplement (high calorie, high protein supplement or low fat/low sugar, high protein supplement) and exercise intervention	Case Comprehensive Cancer Center
NCT02607826	Short-term Starvation	University Hospital Tuebingen

## Data Availability

No new data were created or analyzed in this study.
